# Editorial: Novelties in the therapeutic approaches for chronic heart failure: cardiovascular targets and beyond, volume II

**DOI:** 10.3389/fcvm.2023.1256656

**Published:** 2023-08-02

**Authors:** Massimo Iacoviello, Antonio Sorgente, Andrea Passantino

**Affiliations:** ^1^ Cardiology Unit, Cardiothoracic Department, Polyclinic University Hospital of Bari, Bari, Italy; ^2^ Heart Rhythm Management Center, Postgraduate Program in Cardiac Electrophysiology and Pacing, European Reference Networks Guard-Heart, Universitair Ziekenhuis Brussel-Vrije Universiteit Brussel, Brussels, Belgium; ^3^ Department of Cardiology, EpiCURA, Hornu, Belgium; ^4^ Istituti Clinici Scientifici Maugeri IRCCS, Cardiac Rehabilitation Unit of Bari Institute, Bari, Italy

**Keywords:** heart failure, therapy, congestion, prevention, resynchronisation therapy

**Editorial on the Research Topic** Novelties in the therapeutic approaches for chronic heart failure: cardiovascular targets and beyond, volume II

The second volume of “*Novelties in the Therapeutic Approaches for Chronic Heart Failure: Cardiovascular Targets and Beyond*” aims to further focus on the current and future treatment of chronic heart failure (CHF), a clinical syndrome that, despite recent progress, still has a severe prognosis (Correale et al.). In this complex clinical scenario, the review by Correale et al. summarizes the pathophysiological pathways that could be targets for pharmacological therapy and discusses currently available drugs and those that will be available in the near future. The first part of the review focuses on pharmacological therapy targeting cardiac metabolism, specifically the inhibition of fatty acid uptake by cardiomyocytes, reduction of fatty acid oxidation and circulating levels, and the increase of glucose or ketone oxidation. The authors discuss the potential metabolic effects of drugs such as Perhexiline, Trimetazidine, sodium-glucose cotransporter inhibitors (SGLT2i), and glucagon-like peptide 1 receptor agonists (GLP1RAs), which were initially tested for antidiabetic effects but are now indicated for treating HF patients. The second part of the review is dedicated to novel opportunities to enhance the nitric oxide (NO) pathway, highlighting the positive results of vericiguat in improving the prognosis of HF patients in the VICTORIA-HF trial. Furthermore, the review discusses drugs that act on mitochondrial function, intracellular calcium dysregulation, and cardiac myosin.

In a clinical scenario characterized by a number of possible therapeutic approaches, the identification of features useful for personalizing therapy could play a key role in the future. In this context, the paper by Su et al. evaluates the role of biomarkers in predicting resistance to sacubitril/valsartan (Su et al.). Specifically, the study focuses on different transfer RNAs (tRNA) that reflects actively expressed genes and could represent potential biomarkers. Under certain conditions such as hypoxia, oxidative stress, starvation, and high temperature, tRNAs can be cleaved into tRNA-derived small RNAs (tsRNAs) that can serve specific functions. These tsRNAs are currently being studied as therapeutic targets and markers for diagnosis and prognosis assessment. Using bioinformatics, quantitative real-time PCR (qRT-PCR), and cell-based experiments, the authors aimed to detect tsRNAs associated with resistance to sacubitril/valsartan in a group of patients with myocardial infarction. They defined resistance based on the baseline left ventricular ejection fraction (LVEF) and its changes after drug administration. Patients were defined as resistant if they showed a LVEF <40% or <10% higher than baseline at the last review. The study found that certain tsRNAs were upregulated or downregulated in patients with resistance to sacubitril/valsartan, suggesting their potential utility in evaluating the therapeutic heterogeneity of this drug. In patients with resistance to sacubitril/valsartan the expression of tRF-59:76-Tyr-GTA-2-M3 and tRF-60:76-Val-AAC-1-M5 was upregulated, while the expression of tRF-1:29-Gly-GCC-1 was downregulated. The findings were confirmed by receiver operating characteristic (ROC) curves, with areas under the curves ranging between 0.875 and 0.847. These biomarkers hold promise for helping clinicians tailor therapy in CHF patients.

Personalized therapy will play an even more significant role in patients with heart failure with preserved ejection fraction (HFpEF) which actually characterize more than a half of CHF patients and whose prevalence could further increase in the next years Peh et al. Despite the increasing interest in potential therapeutic targets for HFpEF, there is currently a lack of approaches that significantly improve the prognosis of patients, except for SGLT2i. This is in part due to the heterogeneity of HFpEF population both in terms of etiology and pathophysiology. For this reason, over the last years, the new proposed therapeutic approaches were focused on specific etiologies such as amyloidosis for tafamidis or hypertrofic cardiomyopathy for mevacamten, as well as on specific phenotypes and their related pathophysiological pathways. The review by Peh et al. focuses on one of these pathways, i.e., the inflammation, as a specific therapeutic target for HFpEF (Peh et al.). The concept of “metainflammation” describes the association between metabolic stress caused by diabetes, obesity, insulin resistance, and nonalcoholic fatty liver disease and chronic inflammation, which can lead to adverse cardiac remodeling and HFpEF. The review discusses how existing HF therapies can interfere with inflammation and describes new therapies that target inflammation, offering insights into future phenotype-based therapeutic approaches for HFpEF.

Two papers in the special issue focus on congestion, which is a major factor in HF worsening and hospitalizations. The study by Pathangey et al. evaluates the safety, efficacy, and outcomes of outpatient intravenous diuresis in a rural setting (Pathangey et al.). The trial included CHF patients with worsening congestion and a resting systolic blood pressure above 90 mm Hg. The diuretic infusion regimen in the study was tailored based on the patients' home medications, electrolytes, renal function, and blood pressure. Boluses, drip infusions over several hours, or a combination of both were all possible. No changes in patient's home guideline-directed medical therapy, such as ARNI and SGLT2i were associated. The post-diuresis response was assessed by phone calls at 24–72 h. In this single center study, 60 patients were included. The study demonstrated the feasibility, safety, and effectiveness of providing outpatient IV diuresis, with a 30-day readmission rate comparable to urban outpatient IV centers, suggesting this therapeutic option could be an affordable model for rural HF patients.

The second study on congestion evaluated the role of the lymphatic system in managing fluid overload among HF patients (Li et al.). The lymphatic system plays a crucial role in fluid homeostasis. In their study, Li and coll. compared the Optimal Lymph Flow for Heart Failure (TOLF-HF) program with usual care in 66 HF patients, who were randomized to receive or not the treatment for 4 weeks. The trial demonstrates that TOLF intervention is associated with a reduction in the prevalence of fluid overload symptoms, improvement in abnormal weight gains and physical functions. These results are interesting for different reasons. First of all, TOLF intervention includes self-care strategies to activate the lymphatic system, such as muscle-tightening deep breathing and muscle tightening pumping exercises, as well as large muscle exercises. These exercises activate lymphatic ducts, facilitate lymph fluid drain, help lymph fluid flow and reduce fluid build-up and enhance lymph fluid flow and drain across. The second aspect is related to the pathophysiological consequences of the lymphatic system activation. In fact, the exercise related accelerate fluid volume removal at the level of the thoracic area but also throughout the whole body can allow positive outcomes related to the fluid overload symptom relief. These aspects strengthen the execution of trials on larger samples in order to confirm these results and demonstrate the possible usefulness on end-points related to heart failure progression.

In addition to pharmacological approaches, electrical therapy plays a crucial role in improving outcomes for HF patients. Since the end of nineties, beside to the prevention of sudden death by implantable cardioverter defibrillator, cardiac resynchronization therapy (CRT) based on biventricular pacing (BiV-CRT) has been a fundamental therapeutic strategy in patients affected by heart failure with reduced ejection fraction (HFrEF) and prolonged QRS duration, particularly in those showing a left bundle branch block (LBBB) Zhang et al. The correction of the electromechanical consequences associated to left ventricular asynchrony by BiV-CRT has demonstrated to be greatly effective in terms of reverse remodeling and improvement of patients’ prognosis Zhang et al. However, a novel physiologic CRT approach based on left bundle branch area pacing (LBBaP) has been introduced in recent years. LBBaP offers several advantages over BiV-CRT, including a low and stable pacing capture threshold, high implantation success rate, a short learning curve, and economic feasibility. On the other hand, there is less evidence demonstrating the beneficial effects of LBBaP in comparison with BiV-CRT. Moreover, different conditions could also limit the feasibility of LBBaP such as the presence of non-specific intraventricular conduction defects, the presence of myocardial fibrosis, the impairment of atrial and ventricular functions. The review of Zhang et al. summarizes all the evidence about LBBaP and discuss its advantages and disadvantages (Zhang et al.).

Finally, the paper of Du et al. highlight a novel aspect concerning the best strategy to prevent HF in its preclinical stages. Over the last years, two classes of drugs have shown the ability to reduce this risk in patients with type 2 diabetes (T2D): the GLP1 receptor agonists (GLP1RAs) and the SGLT2 inhibitors (SGLT2is) (Du et al.). A paper of Baviera and coll (1). has recently evaluated the effectiveness of GLP1RAs vs. SGLT2is on cardiovascular and cerebrovascular events in T2D patients. Previous evidence from randomized controlled trials (RCTs) suggested similar benefits of GLP1RAs and SGLT2is in terms of myocardial infarction (MI), major cardiovascular events (MACE), and all-cause mortality (ACM), with GLP1RAs being superior in reducing stroke and SGLT2is in reducing HF hospitalization (HHF). However, the study by Baviera et al. found that GLP1RAs showed significant reductions in the risks of MI and MACE. The risk for HHF was similar between the two drug classes, but in an intention-to-treat analysis, GLP1RAs were associated with a significant reduction in ACM. The results of the study are discussed in the opinion of Du et al., who effectively summarize the previous evidence and delve into the possible reasons for the discrepancy between Baviera's study and previous ones. Factors such as differences in cardiometabolic risk factors in the studied group and the statistical power limitations of the study could be contributing factors. Additionally, the study did not consider that different GLP1RAs and SGLT2is may have different beneficial effects in preventing cardiovascular and cerebrovascular events.

In conclusion, this special issue, like the first one already published (Baviera et al.), presents new and relevant data and opinions that are valuable for all physicians involved in the care of CHF, a complex clinical syndrome whose poor outcome still necessitates every possible effort to enhance our therapeutic options.
